# Smartphone-Assisted Placido Ring Imaging for K1 Stratification in Keratoconus: A Deep Learning Study

**DOI:** 10.3390/diagnostics16132076

**Published:** 2026-07-02

**Authors:** Enes Eroglu, Nicholas Tomaras, Kabir Anand Pathak, Jaron Sanchez, Rafael Alejandro Pinto-Colmenarez, Juan Carlos Prieto, Lucie Dole, Rohith Erukulla, Michael Maizel, Ali R. Djalilian, Mohammad Soleimani

**Affiliations:** 1 Department of Ophthalmology, University of North Carolina at Chapel Hill, 2226 Nelson Highway, Chapel Hill, NC 27517, USA; enes_eroglu@med.unc.edu (E.E.); kabir.pathak@med.unc.edu (K.A.P.); jaronsanchez3@gmail.com (J.S.); pintocol@unc.edu (R.A.P.-C.); jprieto@med.unc.edu (J.C.P.); lucie_dole@med.unc.edu (L.D.); michael_maizel@med.unc.edu (M.M.); 2 Department of Ophthalmology, University of Illinois Chicago, 1855 W. Taylor Street, Chicago, IL 60612, USA; ntomar3@uic.edu (N.T.); reruku2@uic.edu (R.E.); adjalili@uic.edu (A.R.D.)

**Keywords:** keratoconus, keratometry, K1, deep learning, smartphone imaging

## Abstract

**Background/Objectives**: Keratoconus (KC) is a chronic disease that causes progressive corneal thinning and steepening, thereby negatively impacting visual acuity. Although corneal topography and keratometry are the primary measures to diagnose KC, access to these methods can be limited by various factors. To address these limitations, this study evaluates a novel low-cost deep-learning algorithm that infers keratometric categories from smartphone-assisted Placido ring photographs. **Methods**: Development utilized 1240 healthy control eye images and 188 K1-labeled KC images for pretraining, without using their K1 labels. A Variational Autoencoder with KL divergence regularization (AutoEncoderKL) was trained on this pool; its encoder generated latent features for KC images (*n* = 535). A held-out set (*n* = 70) with Pentacam keratometry was labeled by K1 into <40 D, 40–47 D, and >47 D. An ensemble classifier chosen via grid search and cross-validation used the encoder features. Performance was assessed for accuracy, precision, recall, and F1-score. **Results**: The model achieved 91% accuracy across all classes. Precision of the model was 0.77 (<40 D), 0.98 (40–47 D), and 0.86 (>47 D); recall was 0.83, 0.91, and 1.00; and F1-scores were 0.80, 0.94, and 0.92, respectively. Notably, the model achieved perfect recall for the >47 D K1 category. **Conclusions**: A smartphone-assisted Placido ring imaging approach was able to predict K1-based keratometric categories without requiring tomographic or keratometric measurements as model inputs at inference. These findings provide preliminary proof-of-concept for the potential use of smartphone-assisted Placido ring images as a low-cost approach for K1-based stratification. Larger externally validated studies across different sites, devices, operators, printed Placido discs, acquisition conditions, and patient populations are required before clinical utility can be assessed.

## 1. Introduction

Keratoconus (KC) is a progressive eye disorder marked by paracentral corneal thinning and irregular anterior curvature that degrades optical quality and can result in severe visual loss [1,2]. Early detection matters because corneal collagen cross-linking (CXL) can biomechanically stabilize the cornea and reduce the likelihood of progression, with substantial effects reported on long-term follow-up [3]. Globally, the epidemiology of KC is heterogeneous. Recent literature indicates wide variation across regions and study designs, highlighting a substantial and underrecognized burden [4,5,6]. Within the United States, emerging population-based data suggest inequities in both prevalence and disease severity among Black and Hispanic populations, who are disproportionately affected. These patterns have important implications for access and outcomes [4,5,6].

Despite consensus that corneal topography, tomography, and keratometry are the diagnostic standard, these modalities require costly hardware and trained personnel that are rarely available outside specialty settings. These constraints limit timely identification and longitudinal monitoring, especially in resource-limited or rural environments [7,8]. Even artificial intelligence (AI) systems that analyze tomographic outputs require access to such devices [9]. Parallel advances in ophthalmic AI have demonstrated strong performance for anterior-segment tasks, and several groups have begun to explore smartphone-acquired imagery as a pragmatic front end for screening and triage. For example, recent work shows that deep learning (DL) applied directly to smartphone anterior-segment photographs can triage multiple corneal conditions, supporting the feasibility of high-quality analysis without slit-lamp cameras or tomography [10,11,12].

A distinct line of smartphone solutions for KC seeks to recreate topography using clip-on accessories (e.g., 3D-printed Placido discs with LED rings) that project onto the corneal surface. Early studies report promising recall, specificity, and correlation with clinical keratometry. However, these systems reintroduce hardware costs, workflow complexity, and operator training [7,13,14,15]. In contrast, a simplified smartphone-assisted Placido ring approach using an inexpensive, easily reproducible cardboard disc may retain the portability of smartphone-based imaging while minimizing the hardware cost and workflow complexity associated with more elaborate clip-on or illuminated Placido systems.

The biological and optical rationale for this approach is indirect. KC is a corneal ectatic disorder; therefore, iris tissue itself is not expected to encode keratometric pathology [1,7,8]. However, corneal and iris-centered anterior-segment photographs acquired with a Placido ring target provide a standardized spatial frame and may capture optical correlates of corneal steepening, including changes in limbal contour, apparent pupil or iris geometry, peri-corneal reflections, shadows, and local image distortions. Advanced KC may also produce externally visible signs, such as altered light reflexes or cone-related eyelid deformation [1,7,8]. Thus, smartphone-assisted Placido ring photographs centered on the iris and corneal region may contain learnable image features associated with keratometric categories. This relationship is not equivalent to direct measurement of corneal curvature, pachymetry, or posterior elevation; rather, it supports preliminary stratification of corneal steepness using a low-cost and readily accessible imaging approach.

This study evaluates whether a smartphone-assisted Placido ring DL pipeline can infer clinically relevant K1 categories from anterior-segment photographs. Methodologically, iris-centered anterior-segment crops were used to standardize the field of view and capture peri-corneal optical features potentially associated with corneal steepening, without assuming that iris tissue itself encodes keratometric pathology. This approach leverages unsupervised representation learning from a combined pool of smartphone photographs comprising 1240 healthy control eye images from the Soleimani Laboratory at the University of North Carolina at Chapel Hill and 188 K1-labeled KC images, without using K1 labels during pretraining. A variational autoencoder-based representation learning model was trained on this pretraining pool to learn compact latent features from noisy, variable smartphone imagery. After training, the fixed encoder was applied to an independent KC image set labeled with Pentacam keratometry for downstream classification.

Rather than regressing continuous keratometry, the task was cast as three prespecified K1 categories: <40 D, 40–47 D, and >47 D. K1 was selected as an initial proof-of-concept endpoint because it provides a standardized, reproducible keratometric label available from Pentacam imaging and allows model output to be framed as a broad triage category rather than a definitive diagnostic classification. For this proof-of-concept analysis, the thresholds defined lower, intermediate, and higher K1 strata, providing an ordered framework for evaluating the model’s ability to distinguish increasing levels of corneal steepness [16,17,18,19,20]. The resulting latent features were mapped to these K1 categories using a lightweight ensemble classifier. This design reflects a practical trade-off: the pipeline avoids specialized optics at inference while harnessing deep networks and ensemble methods that are proven effective across anterior-segment literature for noisy, variable smartphone imagery [10,11,12].

The objective of this study was to determine whether this low-cost smartphone-assisted Placido ring DL pipeline could accurately stratify K1 categories. By aligning model outputs with an ordered framework of increasing corneal steepness, this approach provides preliminary proof-of-concept for accessible K1-based stratification and establishes a foundation for future evaluation in screening and triage settings.

## 2. Materials and Methods

### 2.1. Study Design and Oversight

A methodological study was implemented to evaluate a smartphone-photo deep learning pipeline for classification of prespecified K1-based keratometric categories. The study adhered to the tenets of the Declaration of Helsinki and was approved by the Institutional Review Board of the University of Illinois Chicago Office for the Protection of Research Subjects (Protocol No. STUDY2023-0883: Keratoconus Registry) on 3 October 2023.

### 2.2. Data Sources and Cohorts

**Figure 1:**
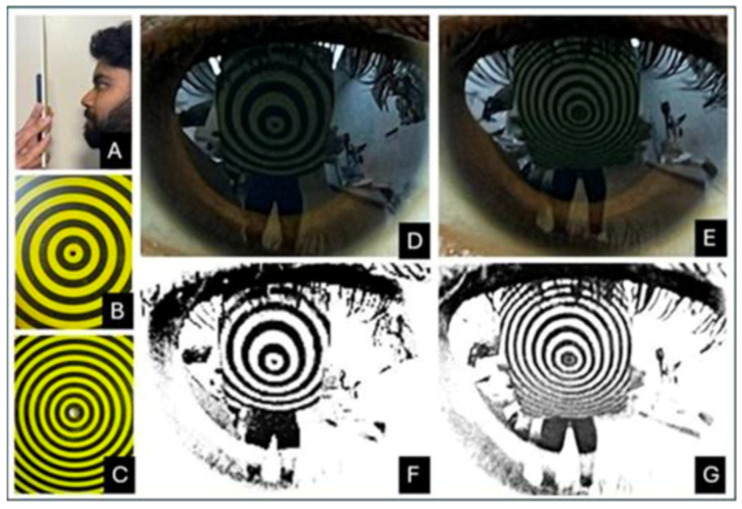
Picture illustrating the technique for capturing images of a Placido disc for the keratoconus dataset. The image displays two different Placido discs (**A**–**C**), highlighting the reflections on the iris (**D**–**G**).

Smartphone anterior-segment photographs were drawn from two independent sources. The primary labeled dataset comprised an independent KC cohort (*n* = 535) containing smartphone iris and eye photographs with associated Pentacam (Oculus, Wetzlar, Germany) keratometry measurements. All anterior-segment photographs were captured with participants looking forward in a standardized gaze position using an iPhone 12 Pro Max (Apple Inc., Cupertino, CA, USA). Images were obtained using a handheld smartphone camera aligned with the central aperture of a rectangular Placido disc measuring approximately 20 cm × 27 cm. The Placido disc was positioned in front of the eye to generate concentric mires, and the camera was aligned with the central opening of the disc (Figure 1). The working distance was approximately 20 cm from the eye and was adjusted slightly by the operator to obtain a focused image with centered Placido reflections on the cornea. No external chin rest, head mount, or mechanical stabilizing apparatus was used. Instead, participants were instructed to maintain forward fixation, and the operator repeated image acquisition when fixation, focus, or ring centration was inadequate. Images with severe blur, occlusion, failed segmentation, or poor image quality were excluded prior to model training or evaluation.

A pool of 347 images from 70 unique eyes was separated at the patient and eye level and reserved exclusively for final evaluation. The remaining 188 KC images, representing 40 eyes, had associated Pentacam-derived K1 labels and were used at two sequential stages of model development. First, these images were incorporated into the AutoEncoderKL (version 0.2.3) pretraining pool, during which their K1 labels were withheld and not used for unsupervised representation learning. After pretraining, the encoder was fixed, and latent features extracted from the same 188 images were used with their K1 labels for supervised classifier training, hyperparameter selection, and five-fold cross-validation; within this development set, 150 images (31 eyes) were used for training-split weight optimization and 38 images (9 eyes) for validation-split hyperparameter monitoring. Together with the 1240 healthy-control images, the 188 KC development images yielded a pretraining pool of 1428 images, which was partitioned using a random 80/20 split into an unsupervised autoencoder training subset (*n* = 1142 images) and an independent validation subset (*n* = 286 images). A comprehensive tracking ledger of unique eyes, image counts, and explicit study assignments across every pipeline phase is provided in Table 1.

The healthy control dataset comprised 1240 smartphone anterior-segment photographs of clinically normal eyes obtained from the Soleimani Laboratory at the University of North Carolina at Chapel Hill. These images were used solely for unsupervised representation learning to improve the generalizability of the autoencoder. Images were captured across multiple device types under varied ambient lighting conditions. No keratometry labels were associated with the healthy control images; they were used for pretraining only.

The unit of analysis varied by pipeline stage. For unsupervised autoencoder pretraining and supervised classifier training, the image was the primary unit of analysis, utilizing all available images per eligible eye to maximize training data volume and exposure to capture variance. Conversely, for final model evaluation, the eye was strictly treated as the primary unit of analysis to align with clinical standards and prevent data leakage. The held-out evaluation cohort was separated at the patient and eye level before autoencoder pretraining or classifier development and initially contained 347 raw images from 70 unique eyes; this cohort was not used for autoencoder pretraining, supervised classifier training, hyperparameter selection, or cross-validation at any stage. Quality-control review excluded 14 images because of severe blur, 9 because of excessive eyelid or other occlusion, 6 because of failed segmentation, and 4 because of other poor-quality acquisition, leaving 314 eligible candidate images across the 70 eyes. Before final image selection, each eye contributed a median of 4 eligible candidate images (interquartile range, 3–6; range, 1–9). For each eye, the single eligible image with the highest iris-segmentation confidence score was retained as the sole representative frame, producing a final eye-level evaluation set of 70 images from 70 unique eyes. All reported performance metrics were calculated exclusively from this final eye-level evaluation set. Patient-level partitioning was enforced prior to any model development, ensuring complete independence between the training and testing cohorts. The primary analysis categorized images by K1 (flat meridian keratometry) into three classes: <40 D, 40–47 D, and >47 D, corresponding to physiologic-range, intermediate, and steeper keratometric categories.

### 2.3. Model Design and Utilization

**Figure 2:**
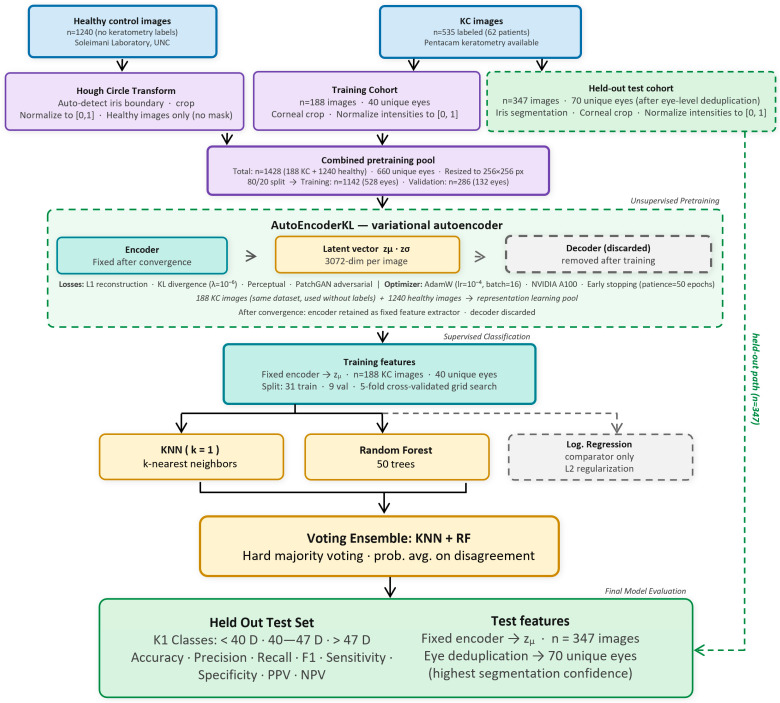
Overview of the K1-category classification pipeline. KC images with segmentation masks and healthy control images without masks undergo parallel preprocessing paths (iris segmentation and Hough Circle Transform, respectively) before merging into a combined pretraining pool (*n* = 1428 total images; split into *n* = 1142 training and *n* = 286 validation images). A Variational Autoencoder with KL divergence regularization (AutoEncoderKL) is trained on this pool; after convergence, the decoder is discarded and the encoder is retained as a fixed feature extractor, producing a compact latent vector (*z_µ_*) per image. These features are used to train and tune three classifiers via 5-fold cross-validated grid search. The final voting ensemble (KNN + RF) is evaluated on a held-out test set of 70 unique eyes. Dashed borders indicate components not included in the final ensemble. KC = keratoconus; KNN = k-nearest neighbors; RF = random forest.

The classification pipeline comprised three sequential stages: iris segmentation and preprocessing, unsupervised representation learning via a variational autoencoder, and supervised classification using the learned latent features (see Figure 2).

#### 2.3.1. Stage 1: Segmentation and Preprocessing

An iris-segmentation model was first applied to each KC smartphone photograph to locate the iris boundary automatically [21,22,23]. This step isolates the anatomically relevant corneal and iris region by removing background structures including eyelids, lashes, sclera, and skin. The segmentation output (a pixel-wise label map) was used to compute a bounding crop centered on the corneal region (label index 2). Pixel intensities were then normalized to the range [0, 1] to standardize brightness and contrast across different cameras and lighting conditions. Images with failed segmentation due to severe blur or heavy occlusion were excluded prior to any model training. During training, all images were augmented with random horizontal axis flip (probability = 0.50) and random rotation up to ±30° (probability = 0.50). No shearing, elastic deformation, or color jitter was applied, to avoid introducing artificially distorted corneal geometry.

For the healthy control images, no pre-existing segmentation masks were available. A Hough Circle Transform was applied to each image to automatically detect the iris boundary circle. Specifically, a Gaussian-blurred grayscale version of each image was processed using the gradient-based Hough accumulator (*dp* = 1, *param*_1_ = 50, *param*_2_ = 30), with minimum and maximum radius constraints set to 10% and 60% of the shorter image dimension, respectively. A padded square crop (padding factor 1.15× detected radius) was extracted around the detected circle center. In cases where no circle was detected, a centered square crop of 40% of the shorter image dimension was applied as a fallback. All images were then resized to 256 × 256 pixels. This approach enabled the healthy control images to be incorporated into representation learning without requiring a dedicated segmentation model.

#### 2.3.2. Stage 2: Representation Learning via Variational Autoencoder

To learn generalizable image features from consumer-grade photographs, a Variational Autoencoder with KL divergence regularization (AutoEncoderKL) was trained on a combined pool of KC images and healthy control images (n_train_ = 1142; n_val_ = 286). The AutoEncoderKL is a convolutional neural network comprising an encoder, a stochastic latent bottleneck, and a decoder [24].

The encoder compresses each 256 × 256 preprocessed image into a compact latent distribution parameterized by a mean vector (*zµ*) and a standard deviation vector (*zσ*). During training, a sample is drawn from this distribution and passed through the decoder to reconstruct the original image. The network is trained jointly with three losses: (i) an L1 pixel-wise reconstruction loss, which penalizes the difference between the reconstructed and original images; (ii) a KL divergence term (weight λ*KL* = 1 × 10^−6^), which regularizes the latent space to follow a unit Gaussian distribution, promoting smooth and continuous feature representations; and (iii) a perceptual loss and patch-based adversarial loss (PatchGAN discriminator), which encourage the decoder to produce perceptually realistic reconstructions rather than blurry pixel averages.

The AutoEncoderKL encoder comprised three convolutional stages with channel widths of 128, 256, and 384, one residual block per stage, and spatial attention applied at the deepest stage. The latent space comprised 3 channels at a spatial resolution of 32 × 32, yielding a 3072-dimensional latent vector per image. Training used the AdamW optimizer with a learning rate of 1 × 10^−4^, weight decay of 0.01, and a batch size of 16. The combined loss weights were: L1 reconstruction (weight = 1.0), KL divergence (λ_KL = 1 × 10^−6^), perceptual loss (weight = 1 × 10^−3^), and PatchGAN adversarial loss (weight = 1 × 10^−2^). The model was trained on the Longleaf high-performance computing cluster (University of North Carolina at Chapel Hill) using an NVIDIA A100 GPU. After convergence, the decoder was discarded. The encoder was retained as a fixed feature extractor: for each preprocessed image, it produces the latent mean vector zμ, which serves as the compact numeric representation (latent feature vector) used in all downstream classification. Perceptual and adversarial loss components were activated after a 10-epoch warm-up. Training ran for up to 200 epochs with early stopping (patience = 50 epochs) monitoring validation reconstruction loss, implemented in Python 3.12.4 using PyTorch Lightning (version 2.2.1), MONAI Generative Models, and scikit-learn. Fixed random seeds were not applied to the stochastic training process; this is acknowledged as a reproducibility limitation, and future experiments should specify seeds for full replication.

Claude Sonnet 4.6 (Anthropic, San Francisco, CA, USA) was used to assist with selected programming code drafting and technical description wording. All AI-assisted code outputs and technical descriptions were reviewed and validated by the authors.

#### 2.3.3. Stage 3: Supervised Classification

Prior to KNN classification, latent feature vectors (*zµ*) were standardized (zero mean, unit variance) across the training set, and the same scaling parameters were applied to validation and held-out test features to avoid leakage. Using the training and validation split of the labeled KC dataset, a grid search with five-fold cross-validation to select hyperparameters for three candidate predictive models was conducted: (i) k-Nearest Neighbors (KNN): k ∈ [1, 24], optimal k = 1; (ii) Random Forest (RF): number of trees ∈ {5, 10, 50, 100, 200, 1000}, optimal = 50; (iii) Logistic Regression (LR): maximum iterations 10,000 with L2 regularization (evaluated as a comparator). Standalone and ensemble performance for all candidate models on the held-out test set is reported in Supplementary Table S1. The selection of k = 1 reflects, in part, the structured geometry of the VAE latent space. KL divergence regularization encourages a smooth, approximately Gaussian latent distribution in which nearest-neighbor queries may correspond to morphologically similar images. However, this explanation does not eliminate the risk of overfitting or sample-specific nearest-neighbor behavior, particularly given the high dimensionality of the latent vector relative to the small, supervised training set (*n* = 188 images, 40 eyes). Latent features were standardized prior to KNN classification to mitigate scale-driven distance distortion. The final ensemble combined the tuned KNN (*k* = 1) and RF (50 trees) via hard majority voting. For each image, if both models predicted the same K1 class, that label was assigned. In cases of disagreement, the unweighted average of their class probability vectors was computed and the class with the highest average probability was selected. Logistic Regression was retained as a secondary comparator but excluded from the ensemble, as its linear decision boundary is likely to underfit the nonlinear latent space produced by the autoencoder.

### 2.4. Outcomes and Evaluation

**Figure 3:**
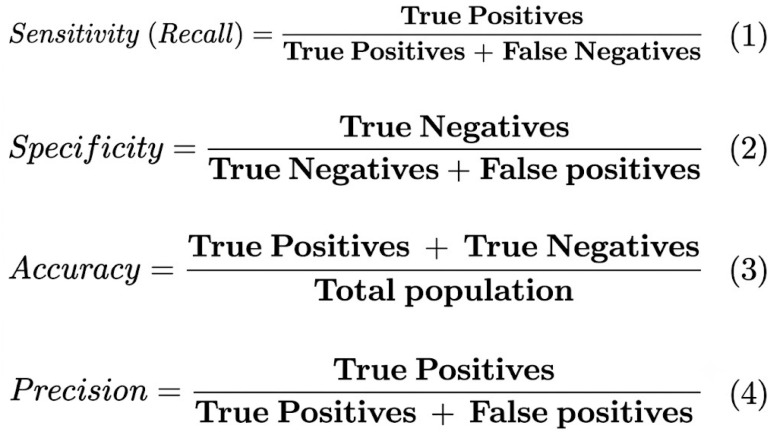
Definitions of evaluation metrics.

All splits were created at the subject and eye level to prevent information leakage between training and evaluation sets. The held-out test set (*n* = 70 unique eyes) with Pentacam keratometry was not used for fitting, tuning, or model selection and was accessed only once for final evaluation. The primary endpoint was overall accuracy for the three-class K1 categorization task. Secondary endpoints were per-class precision, recall, F1-score, macro-averaged metrics, and a normalized confusion matrix. Metric definitions follow standard formulas (Figure 3).

## 3. Results

Table 2 provides a comprehensive report of the per-class precision, recall, and F1-scores for the three defined K1 categories. These metrics are accompanied by macro and support-weighted averages, which offer a high-level view of model performance across the dataset. Precision and recall are accompanied by 95% Wilson confidence intervals, calculated from the per-class true positive, false positive, and false negative counts. Macro-averaged and weighted-averaged precision, recall, and F1-score, along with the per-class F1-scores themselves, are composite statistics derived from these underlying class-level metrics and are reported as point estimates only. On the held-out test set (*n* = 70 eyes), the ensemble model demonstrated robust performance, achieving an overall accuracy of 91%. The balanced accuracy, defined as the unweighted mean of per-class recall, was 0.913, confirming that overall performance was not disproportionately inflated by the center class. Notably, the model achieved perfect recall (1.00) in the upper tail class (>47 D). Precision and F1-scores reached their peak in the central class (40–47 D), while the lower tail class (<40 D) exhibited the lowest values. The macro-F1 and weighted-F1 values serve to summarize the model’s balanced performance, accounting for the inherent class distribution variance within the test subset.

Exploratory binary K1-threshold analyses were also performed. For classification of K1 ≥ 40 D versus <40 D, the model achieved a sensitivity of 0.948 (95% CI: 0.86–0.98) and specificity of 0.833 (95% CI: 0.55–0.95), with a PPV of 0.965 and NPV of 0.769. For classification of K1 > 47 D versus ≤47 D, the model achieved a sensitivity of 1.000 (95% CI: 0.76–1.00) and specificity of 0.966 (95% CI: 0.88–0.99), with a PPV of 0.857 and NPV of 1.000. These results indicate that no eyes with K1 > 47 D were missed within this small and enriched held-out population, and all eyes within this highest K1 category were correctly identified (Table 3).

**Figure 4:**
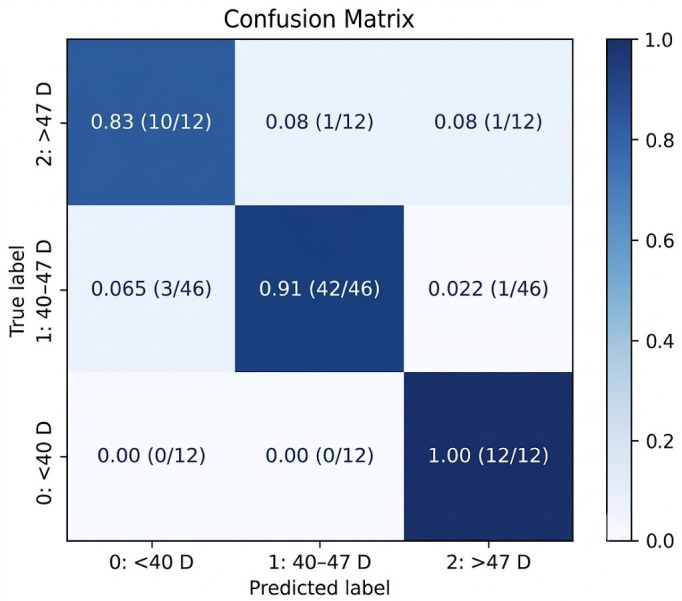
Normalized confusion matrix on the test subset (*n* = 70) for K1-based three-class classification: 0 = <40 D, 1 = 40–47 D, 2 = >47 D. Rows = true class; columns = model’s predicted class. Each cell reports the row-normalized proportion with the corresponding raw count in parentheses.

The normalized confusion matrix, illustrated in Figure 4, provides a more granular view of the model’s decision-making process. The data reveals a predominantly adjacent-category error pattern, which is critical for understanding the model’s clinical safety profile. Specifically: (i) among the true <40 D images, 83% (10/12) were correctly identified, with 8.3% (1/12) assigned to the 40–47 D class and 8.3% (1/12) assigned to the >47 D class; (ii) for the true 40–47 D cohort, 91% (42/46) were accurately classified, while 6.5% (3/46) were labeled as <40 D and 2.2% (1/46) were labeled as >47 D; (iii) most significantly, all >47 D images (12/12) were correctly identified, yielding the aforementioned perfect recall for severe keratometric steepening.

Thus, most misclassifications occurred between adjacent K1 categories. Limited cross-extreme confusion was observed, with one <40 D image assigned to >47 D, while no >47 D images were assigned to <40 D. Consistent with this pattern, the central class achieved precision of 0.98 and recall of 0.91, whereas the >47 D class achieved perfect recall with precision of 0.86. The <40 D class demonstrated precision of 0.77 and recall of 0.83. This behavior is expected given the class imbalance and the clinical continuity around the 40–47 D threshold. Taken together, the ensemble provides high overall accuracy of 91% and strong class-specific performance on the held-out subset. Errors were concentrated near the intermediate 40–47 D category rather than occurring predominantly between distant categories.

## 4. Discussion

Deep learning (DL) has emerged as a cornerstone of modern medical image analysis, largely due to its capacity to learn complex, hierarchical features directly from pixel-level data. When this capability is paired with rigorous data curation and robust evaluation protocols, DL can uncover patterns that may be imperceptible to human observers [25]. Within the field of ophthalmology, DL has already demonstrated transformative success in posterior-segment applications, such as diabetic retinopathy and glaucoma screening, and is now being increasingly leveraged for anterior-segment pathologies [26]. A particularly promising development is the use of consumer-grade cameras and handheld devices for disease recognition, which highlights the feasibility of low-cost acquisition and decentralized tele-ophthalmology workflows [10,11,27]. Recent smartphone-based research in corneal infection has shown that portable imaging, when integrated with DL pipelines, can provide high-fidelity discrimination, potentially extending specialized ophthalmic care to regions lacking slit-lamp biomicroscopes or dedicated specialty cameras [28].

This research presents a low-cost, smartphone-assisted Placido ring DL approach that predicts keratometric categories from anterior-segment photographs. Using unsupervised representation learning on a large pool of iris and eye images and a supervised ensemble classifier, this model achieved 91% accuracy on a test subset (*n* = 70) across three K1 classes (<40 D, 40–47 D, >47 D) that are clinically significant to KC development [18]. Class-wise behavior was both strong and clinically coherent. The >47 D class achieved perfect recall, with no eyes in the highest K1 category missed in the held-out subset. Most misclassifications occurred between adjacent K1 categories rather than between clinically distant groups, with only limited cross-extreme confusion observed. These findings suggest that the model learned image features aligned with clinically meaningful K1 boundaries while operating without corneal topographic or tomographic measurements at inference. Together, they support the preliminary feasibility of smartphone-assisted Placido ring imaging for low-cost K1-based stratification in settings where conventional corneal imaging resources are limited. While k = 1 carries some theoretical risk of overfitting in unstructured feature spaces, the KL divergence regularization of the AutoEncoderKL is intended to constrain the latent manifold toward a smoother, approximately Gaussian structure in which local proximity may be more stable than in an unregularized space. This plausible explanation is offered as a partial account for the cross-validated preference for k = 1, but is not independently verified in this study (see Supplementary Table S1 for standalone model comparisons).

Prior KC AI studies frequently trained on topography or tomography outputs, which requires access to expensive platforms, thereby limiting impact in low-resource clinics [7,8,9]. Systematic reviews of AI for KC report high accuracy for disease diagnosis, but also substantial methodological concerns. These factors include heterogeneity across studies, risk of bias from case–control designs, and applicability concerns due to most models depending on device-derived features that may not transfer across hardware or populations [7,8,9].

In contrast, the low-cost smartphone-assisted Placido ring approach used in this study reduces the equipment requirements for image acquisition while maintaining encouraging performance for K1-based stratification. The use of readily available smartphones and inexpensively produced Placido ring targets may facilitate broader investigation of corneal steepness classification in settings where conventional imaging resources are limited. Keratoconus is a chronic, progressive condition where the timing of diagnosis is the primary determinant of long-term visual prognosis; however, the current diagnostic gold standards such as Scheimpflug-based tomography are often inaccessible due to their high capital cost and the need for specialized technical staff [1,7,20]. This screening gap, particularly in rural or resource-constrained regions where patients may not receive a specialized corneal evaluation, can lead to the disease reaching an advanced stage, potentially requiring invasive procedures. The model’s performance in the highest K1 category suggests that predictions above the >47 D threshold may be useful for future investigation of approaches that prioritize patients for tomographic confirmation and specialist evaluation. The predominance of adjacent-category errors suggests that classification uncertainty was concentrated near category boundaries. Correspondingly, limited confusion between the lowest and highest K1 categories indicates that the model rarely confused the extremes of corneal steepness within the held-out subset. This pattern supports future evaluation of a tiered referral workflow in which higher-risk predictions prompt definitive tomographic evaluation, while intermediate predictions identify patients who may benefit from additional assessment or longitudinal follow-up. Rather than replacing the diagnostic precision of established clinical instruments, such an approach could potentially broaden access to preliminary stratification and support more targeted use of advanced diagnostic resources. These findings provide a preliminary foundation for prospective external studies evaluating whether this accessible approach can contribute to community screening or remote triage across different populations, devices, operators, acquisition environments, and printed Placido ring targets.

The implementation of a Variational Autoencoder (VAE) within this pipeline was intended to mitigate some of the noise and variability inherent to smartphone-acquired anterior-segment imagery. Rather than mapping high-dimensional raw inputs directly to discrete labels, the VAE uses unsupervised representation learning to map photographs into a compressed, continuous latent space. By training the encoder–decoder framework on a pool of approximately 1200 unlabeled ocular images prior to supervised classification, the model may learn compact representations of recurring anterior-segment structures, including the corneal limbus and Placido disc reflections, while reducing sensitivity to some sources of variation such as ambient lighting or minor gaze deviation. The inclusion of a KL divergence term encourages a smoother latent manifold, which may reduce memorization of training-specific detail relative to an unregularized autoencoder; however, this has not been formally tested via ablation in this study and should not be interpreted as a guarantee against overfitting. We do not claim that this approach produces hardware-invariant features or a comprehensive representation of ocular geometry. Whether the learned latent space generalizes across devices, operators, acquisition settings, and printed Placido ring targets remains to be confirmed through external validation and dedicated ablation studies comparing the VAE-based pipeline to alternative or non-regularized feature-extraction approaches. Furthermore, the model’s particular strengths include remote imaging and enforcing subject eye-level separation through the preservation of a held-out subset for final testing, aligning with best practices for diagnostic AI in terms of leakage-aware evaluation [9]. Although the central class (40–47 D) did not achieve the highest recall, it achieved the highest precision and F1-score across categories. This suggests that there is stable performance in the clinically ambivalent intermediate range. Furthermore, the highest K1 category (>47 D) achieved perfect recall. This is important clinically, as missing advanced forms of the disease may delay referral and eventual invasive intervention such as corneal transplantation. Most errors occurred between adjacent severity categories, with limited cross-extreme confusion, suggesting that the model distinguished clinically distant categories more reliably than borderline cases.

### Limitations and Future Directions

Several limitations constrain interpretation of these findings. The held-out test subset was small and imbalanced, with 70 eyes distributed across <40 D, 40–47 D, and >47 D categories as 12, 46, and 12 eyes, respectively. This imbalance limits the precision of class-specific estimates, particularly for the two tail classes, and may contribute to the observed center-seeking misclassification pattern. The relatively high overall accuracy may therefore overestimate performance in a more balanced or clinically heterogeneous population.

Furthermore, the dataset was sourced from a single institution, which restricts our ability to assess the model’s generalizability across diverse clinical environments. External factors such as varying smartphone hardware, ambient lighting, patient gaze fixation, and positioning could all impact performance in ways not captured in this study. In real-world community screening settings, additional variability may arise from differences in how Placido discs are printed, cut, mounted, or aligned, and ensuring consistent ring spacing and contrast across low-cost printed discs may present a practical implementation challenge.

It is possible the model has learned certain site-specific or acquisition-specific artifacts rather than universal optical correlates of corneal steepening. In addition, the study’s preprocessing and evaluation workflow may have favored technically cleaner images. Images with substantial blur, occlusion, failed segmentation, or poor acquisition quality were excluded, and for the final eye-level evaluation set, the single eligible image with the highest iris-segmentation confidence score was retained for each eye. Although this approach helped standardize the proof-of-concept analysis and ensured that each eye was represented by an interpretable image, it may have preferentially selected better-centered, better-focused, and higher-quality photographs than would be obtained in unsupervised community screening. Therefore, the reported performance may be optimistic relative to real-world deployment conditions.

Another limitation is that the reported metrics were obtained from a single stochastic execution of the full pipeline. Although the held-out test set was separated at the patient and eye level before model development, fixed random seeds were not applied during random data partitioning, model optimization, or augmentation. As a result, exact replication of the data splits and augmented training samples would not be expected from an independent rerun of the pipeline. The performance estimates should therefore be interpreted as preliminary proof-of-concept results from one model run rather than as a fully characterized measure of run-to-run stability. Repeated seeded experiments would be needed to quantify how much the reported accuracy and class-specific metrics vary across independent runs.

The use of Pentacam K1 values as a ground truth, condensed into three discrete categories, represents another simplification. While this approach was useful for an initial proof-of-concept triage model, keratoconus is a multidimensional ectatic disease that cannot be fully characterized by the flat meridian alone. Early or subclinical keratoconus may manifest through asymmetric astigmatism, localized cone formation, posterior elevation abnormalities, pachymetric thinning, or increased Kmax before substantial changes in K1 are present. Therefore, a K1-based model may have limited sensitivity for early ectasia or forme fruste disease and should not be interpreted as a replacement for comprehensive corneal tomography. Clinically, the rationale for using K1 in this preliminary study was to provide a standardized, reproducible, and broadly interpretable keratometric endpoint for evaluating whether smartphone-acquired anterior-segment images contain features associated with corneal steepening. We also note that the model was not directly benchmarked against human graders within this specific dataset. However, prior work in anterior-segment AI has suggested that DL can match or even exceed clinician performance when using portable imaging for similar tasks [9,10,11].

Looking forward, multi-site validation across diverse ethnicities, devices, and acquisition settings is essential, as evaluating models on independent external data remains a critical benchmark for proving true clinical generalizability [29]. Future studies should compare triage performance of this model to clinician assessment accuracy, as well as quantify workflow impact in terms of quicker and improved patient outcomes. Methodologically, the next step is to refine and broaden the label schema so that clinical risk is more accurately assessed. For example, extending reach from 3-class to 4-class severity (e.g., 47–55 D, ≥55 D) would better reflect KC staging and management pathways, aligning with contemporary clinical consensus that artificial intelligence architectures must adapt to handle nuanced staging and track high-risk progression metrics [30,31]. Furthermore, incorporating regression targets (K1, K2, Kmax) may further improve prioritization while maintaining a low-cost footprint. Implementing these next steps will clarify whether smartphone pipelines can deliver clinically meaningful triage without the setup and staffing burdens of device-dependent systems.

This work demonstrates preliminary evidence that a carefully engineered smartphone pipeline can stratify corneal steepening with high accuracy and clinically sensible error patterns. This approach may offer a feasible path toward low-cost keratoconus triage where tomographers are scarce, but it should not be considered a validated screening tool or a replacement for corneal tomography. Rigorous external validation across larger, more diverse populations, acquisition settings, devices, operators, and printed Placido ring targets is needed to establish generalizability and clinical utility.

## Figures and Tables

**Table 1:** Data partition and pipeline utilization summary.

Pipeline Phase/Cohort Subset	Data Level	Unique Eyes (*N*)	Total Images (*n*)	Specific Role
Global Raw Data Sourced				
Healthy Control Pool	Raw	620	1240	Unsupervised Pretraining Base
Keratoconus Pool	Raw	110	535	Supervised & Evaluation Base
1. Unsupervised Pretraining	Raw (image-level)	660	1428	AutoEncoderKL Training
Training Split	Raw (image-level)	528	1142	Weights updating optimization
Validation Split	Raw (image-level)	132	286	Hyperparameter/Loss monitoring
2. Supervised Classification	Raw (image-level)	40	188	Classifier Training/5-Fold CV
Training Split	Raw (image-level)	31	150	Weights updating optimization
Validation Split	Raw (image-level)	9	38	Hyperparameter/Loss monitoring
3. Final Model Evaluation				Held-Out Evaluation Pipeline
Raw held-out pool	Raw (image-level)	70	347	Pre-QC, pre-deduplication
Excluded: severe blur	Excluded	–	14	Quality-Control Exclusion
Excluded: occlusion	Excluded	–	9	Quality-Control Exclusion
Excluded: failed segmentation	Excluded	–	6	Quality-Control Exclusion
Excluded: poor quality	Excluded	–	4	Quality-Control Exclusion
Eligible candidate images	Post-QC (image-level)	70	314	Median 4 images per eye (IQR 3–6; range 1–9)
Final eye-level test set	Analytic eye-level	70	70	Held-Out Evaluation Only

Note: Rows labeled ‘Raw’ reflect image- or eye-level counts within each processing pool prior to per-eye deduplication. Only the Final Model Evaluation row reflects the analytic, deduplicated eye-level sample used for reported performance metrics.

**Table 2:** Classification performance on the held-out test subset (*n* = 70) by K1 category.

Class/Summary	Support	Precision (95% CI)	Recall (95% CI)	F1-Score
Class 0: <40 D	12	0.77 (0.50–0.92)	0.83 (0.55–0.95)	0.80
Class 1: 40–47 D	46	0.98 (0.88–1.00)	0.91 (0.80–0.97)	0.94
Class 2: >47 D	12	0.86 (0.60–0.96)	1.00 (0.76–1.00)	0.92
Balanced Accuracy	-	-	-	0.91
Macro Average	–	0.87	0.92	0.89
Weighted Average	–	0.92	0.91	0.92

**Table 3:** Exploratory binary K1-threshold classification performance on the held-out test subset (*n* = 70 eyes).

K1 Threshold	Sensitivity (95% CI)	Specificity (95% CI)	Positive Predictive Value (PPV)	Negative Predictive Value (NPV)
K1 ≥ 40 D versus <40 D	0.948 (0.86–0.98)	0.833 (0.55–0.95)	0.965	0.769
K1 > 47 D versus ≤47 D	1.000 (0.76–1.00)	0.966 (0.88–0.99)	0.857	1.000

## Data Availability

The original contributions presented in the study are included in the article, further inquiries can be directed to the corresponding authors.

## Supplementary Materials

The following supporting information can be downloaded at: https://www.mdpi.com/article/10.3390/diagnostics16132076/s1, Table S1. Standalone and ensemble model performance on the held-out test set (*n* = 70 eyes).

## References

[1] Singh R.B., Koh S., Sharma N., Woreta F.A., Hafezi F., Dua H.S., Jhanji V. (2024). Keratoconus. Nat. Rev. Dis. Primers.

[2] Bui A.D., Truong A., Pasricha N.D., Indaram M. (2023). Keratoconus diagnosis and treatment: Recent advances and future directions. Clin. Ophthalmol..

[3] Wittig-Silva C., Chan E., Islam F.M., Wu T., Whiting M., Snibson G.R. (2014). A randomized, controlled trial of corneal collagen cross-linking in progressive keratoconus: Three-year results. Ophthalmology.

[4] Singh R.B., Parmar U.P.S., Jhanji V. (2024). Prevalence and economic burden of keratoconus in the United States. Am. J. Ophthalmol..

[5] Christensen M., Kartchner J., Giegengack M., Thompson A.C. (2024). A comparison of Black and non-Black patients in the presentation and treatment of keratoconus. Clin. Ophthalmol..

[6] Nugent L., Son H.S., Wang J.M., Varadaraj V., Smith K., Soiberman U.S., Srikumaran D. (2024). Racial variation in visual impairment of patients with keratoconus at presentation. Cornea.

[7] Abou Said G.A., Gispets J., Shneor E. (2025). Strategies for early keratoconus diagnosis: A narrative review of evaluating affordable and effective detection techniques. J. Clin. Med..

[8] Zhang X., Munir S.Z., Karim S.A.S., Munir W.M. (2021). A review of imaging modalities for detecting early keratoconus. Eye.

[9] Vandevenne M.M., Favuzza E., Veta M., Lucenteforte E., Berendschot T.T., Mencucci R., Nuijts R.M.M., Virgili G., Dickman M.M. (2023). Artificial intelligence for detecting keratoconus. Cochrane Database Syst. Rev..

[10] Maehara H., Ueno Y., Yamaguchi T., Kitaguchi Y., Miyazaki D., Nejima R., Inomata T., Kato N., Chikama T.-I., Ominato J. (2025). Artificial intelligence support improves diagnosis accuracy in anterior segment eye diseases. Sci. Rep..

[11] Ting D.S.J., Foo V.H., Yang L.W.Y., Sia J.T., Ang M., Lin H., Chodosh J., Mehta J.S., Ting D.S.W. (2021). Artificial intelligence for anterior segment diseases: Emerging applications in ophthalmology. Br. J. Ophthalmol..

[12] Jin K., Grzybowski A. (2025). Advancements in artificial intelligence for the diagnosis and management of anterior segment diseases. Curr. Opin. Ophthalmol..

[13] Askarian B., Askarian A., Tabei F., Chong J.W. (2025). An IoT-enabled mHealth sensing approach for remote detection of keratoconus using smartphone technology. Sensors.

[14] Hashemi H., Doroodgar F., Niazi S., Khabazkhoob M., Heidari Z. (2024). Comparison of different corneal imaging modalities using artificial intelligence for diagnosis of keratoconus: A systematic review and meta-analysis. Graefes Arch. Clin. Exp. Ophthalmol..

[15] Huerta-Carranza O., Campos-García M., Moreno-Oliva V.I., Aguirre-Aguirre D., Pérez-Lomelí J.S. (2022). Smartphone-based corneal topography with null-screens. Appl. Opt..

[16] Ganesh D., Lin S.R. (2023). Global metrics on ocular biometry: Representative averages and standard deviations across ten countries from four continents. Eye.

[17] Kamiya K., Ishii R., Shimizu K., Igarashi A. (2014). Evaluation of corneal elevation, pachymetry and keratometry in keratoconic eyes with respect to the stage of Amsler-Krumeich classification. Br. J. Ophthalmol..

[18] Naderan M., Shoar S., Kamaleddin M.A., Rajabi M.T., Naderan M., Khodadadi M. (2015). Keratoconus clinical findings according to different classifications. Cornea.

[19] Kamiya K., Kono Y., Takahashi M., Shoji N. (2018). Comparison of simulated keratometry and total refractive power for keratoconus according to the stage of Amsler-Krumeich classification. Sci. Rep..

[20] Owusu S., Zaabaar E., Kwarteng M.A., Ankamah S., Abowine J.B.V., Kyei S. (2023). Scheimpflug-derived keratometric, pachymetric and pachymetric progression indices in the diagnosis of keratoconus: A systematic review and meta-analysis. Clin. Ophthalmol..

[21] Bazrafkan S., Thavalengal S., Corcoran P. (2018). An end-to-end deep neural network for iris segmentation in unconstrained scenarios. Neural Netw..

[22] Arsalan M., Naqvi R.A., Kim D.S., Nguyen P.H., Owais M., Park K.R. (2018). IrisDenseNet: Robust iris segmentation using densely connected fully convolutional networks in the images by visible light and near-infrared light camera sensors. Sensors.

[23] Prieto J.C., Shah H., Jones K.R., Chew R.F., Kana H.M., Weaver J., Flueckiger R.M., McPherson S., Gower E.W. Image sequence analysis via GRU and attention for trachomatous trichiasis classification. Proceedings of the Fourth Conference on Medical Imaging with Deep Learning, PMLR.

[24] Rombach R., Blattmann A., Lorenz D., Esser P., Ommer B. High-resolution image synthesis with latent diffusion models. Proceedings of the IEEE/CVF Conference on Computer Vision and Pattern Recognition (CVPR).

[25] Shen D., Wu G., Suk H.I. (2017). Deep learning in medical image analysis. Annu. Rev. Biomed. Eng..

[26] Ting D.S.W., Peng L., Varadarajan A.V., Keane P.A., Burlina P.M., Chiang M.F., Schmetterer L., Pasquale L.R., Bressler N.M., Webster D.R. (2019). Deep learning in ophthalmology: The technical and clinical considerations. Prog. Retin. Eye Res..

[27] Fang X., Deshmukh M., Chee M.L., Soh Z.-D., Teo Z.L., Thakur S., Goh J.H.L., Liu Y.-C., Husain R., Mehta J.S. (2022). Deep learning algorithms for automatic detection of pterygium using anterior segment photographs from slit-lamp and hand-held cameras. Br. J. Ophthalmol..

[28] Wang L., Chen K., Wen H., Zheng Q., Chen Y., Pu J., Chen W. (2021). Feasibility assessment of infectious keratitis depicted on slit-lamp and smartphone photographs using deep learning. Int. J. Med. Inform..

[29] Chen X., Zhao J., Iselin K.C., Borroni D., Romano D., Gokul A., McGhee C.N.J., Zhao Y., Sedaghat M.-R., Momeni-Moghaddam H. (2021). Keratoconus detection of changes using deep learning of colour-coded maps. BMJ Open Ophthalmol..

[30] Ferdi A.C., Nguyen V., Gore D.M., Allan B.D., Rozema J.J., Watson S.L. (2019). Keratoconus natural progression: A systematic review and meta-analysis of 11,529 eyes. Ophthalmology.

[31] Goodman D., Zhu A.Y. (2024). Utility of artificial intelligence in the diagnosis and management of keratoconus: A systematic review. Front. Ophthalmol..

