# Supplementary material for: Smartphone-Assisted Placido Ring Imaging for K1 Stratification in Keratoconus: A Deep Learning Study
Source: Diagnostics (Basel). 2026 Jul 2;16(13):2076. doi: 10.3390/diagnostics16132076 (PMC13360261; doi:10.3390/diagnostics16132076)
Supplement: Supplementary file 1 [file diagnostics-16-02076-s001.zip › diagnostics-4350836-supplementary.pdf]

Supplementary Material

Table S1. Standalone and ensemble model performance on the held-out test set (n=70 eyes).

Model	Balanced Accuracy	Macro Average F1-Score	Weighted Average F1-Score
KNN (k=1)	0.857	0.823	0.851
Random Forest (50 trees)	0.871	0.839	0.866
Logistic Regression	0.800	0.751	0.795
Final Ensemble (KNN + RF)	0.913	0.887	0.915
